# A Nasal Taxifolin Hydrogel Targets the TLR4/NF-κB/HIF-1α Axis to Suppress Ferroptosis in Alzheimer’s Disease

**DOI:** 10.3390/antiox15030316

**Published:** 2026-03-03

**Authors:** Miao Zhang, Liangliang Zhu, Yusu Wang, Weijia Chen, Zhongmei He

**Affiliations:** College of Chinese Medicinal Materials, Jilin Agricultural University, Changchun 130118, China; zhmiao96@163.com (M.Z.); zhulliang2015@163.com (L.Z.); jlauwang@163.com (Y.W.); chenweijia_jlau@163.com (W.C.)

**Keywords:** ferroptosis, mitochondrial dysfunction, taxifolin, Alzheimer’s disease, nasal brain delivery system

## Abstract

In order to further explore new therapeutic targets for Alzheimer’s disease (AD), this study, under the guidance of network pharmacology and molecular docking analysis, focused on the TLR4/NF-κB/HIF-1α signal axis and ferroptosis and verified the mechanism of a nasal taxifolin thermosensitive hydrogel (TF-Gel). In the Okada acid (OA)-induced AD mouse model, intranasal administration of TF-Gel significantly improved cognitive dysfunction and reduced neuroinflammation and oxidative stress. Mechanism studies have shown that TF-Gel effectively reduces the accumulation of reactive oxygen species in the hippocampus, enhances mitochondrial membrane potential, and improves mitochondrial ultrastructure by specifically inhibiting the TLR4/NF-κB/HIF-1α pathway, thereby effectively inhibiting neuronal ferroptosis. Western blot analysis confirmed the regulation of ferroptosis, synaptic function, and apoptosis-related proteins by TF-Gel. Of particular importance, the therapeutic benefits of TF-Gel were completely abolished by co-administration of the ferroptosis inducer Erastin, directly confirming that ferroptosis inhibition is the core link in its neuroprotective effect. This study reveals for the first time that TF-Gel exerts a multi-target neuroprotective effect by precisely regulating the TLR4/NF-κB/HIF-1α axis ferroptosis pathway, providing a new perspective for research into the mechanism and treatment of AD.

## 1. Introduction

Alzheimer’s disease (AD) is a chronic neurodegenerative disorder characterized by a prolonged disease trajectory and an inability to be cured [1,2]. The pathogenesis of AD is complex and involves multiple regulatory pathways, with extensive investigations into its pathological mechanisms [3,4]. The hallmark features of AD include the accumulation of β-amyloid protein (Aβ) and the hyperphosphorylation of tau protein, leading to the formation of neurofibrillary tangles. These features have historically dominated research into the disease’s pathology [5,6]. However, the clinical translation of treatment strategies based on these classical pathological mechanisms is fraught with challenges and adverse effects [7,8]. Recent studies have illuminated the intricate nature of AD’s pathogenesis, revealing it to be more complex than traditionally understood, characterized by multi-level and multi-system cellular dysfunction [9,10]. Researchers have increasingly shifted their focus from a sole emphasis on protein toxicity to a broader consideration of cellular homeostasis imbalances. Within this context, ferroptosis and mitochondrial dysfunction have emerged as interrelated mechanisms of cell death that are regarded as fundamental contributors to the progressive neuronal loss observed in AD [11,12].

Ferroptosis is a novel regulatory cell death mode driven by lipid peroxidation, which is highly consistent with the exacerbation of oxidative stress damage in AD, the depletion of antioxidant defense systems (such as the glutathione system), and abnormal brain iron metabolism [13,14]. At the same time, as the core cellular functional center for energy metabolism and reactive oxygen species generation, mitochondrial dysfunction is not only an early and persistent pathological feature of AD, but also a key upstream event that drives and exacerbates oxidative stress and lipid peroxidation product accumulation, thereby triggering the cascade reaction of ferroptosis [15,16]. These two mechanisms are interrelated and synergistically promote ferroptosis in the oxidative stress process driven by mitochondrial damage, which further deteriorates mitochondrial function. The two form a vicious cycle, constituting the core axis of AD pathological progression, directly leading to and accelerating the degenerative development of neurons [17,18].

Taxifolin (TF) is a natural active molecule mainly derived from citrus fruits and milk thistle. It not only has excellent antioxidant capacity, but also exhibits various neuroprotective activities, such as anti-inflammatory properties and the inhibition of cell apoptosis. Therefore, taxifolin has shown important potential in the prevention and treatment of neurological diseases [19,20]. It is worth mentioning that taxifolin has been listed as a new food ingredient, belonging to the category of “medicinal and edible homology”, with high safety. The European Food Safety Authority has confirmed that taxifolin is a safe food ingredient and can be used as a food additive [21]. However, although previous studies have suggested that taxifolin has significant effects in anti-AD treatment [22,23], its underlying mechanism of action still needs to be systematically elucidated. As advancements in big data technology progress, network pharmacology has emerged as a powerful tool for drug research. This approach enables a comprehensive exploration of disease mechanisms and the modes of action of therapeutic agents from a holistic biological network perspective.

The conventional treatment paradigm for neurological diseases primarily relies on oral administration or direct intracerebral injections. However, these approaches frequently encounter challenges posed by the blood–brain barrier and systemic circulation, leading to suboptimal drug delivery efficiency, the dilution of drug concentrations in the bloodstream, and diminished therapeutic efficacy [24,25]. Direct injection techniques, on the other hand, present significant safety concerns and are often associated with notable side effects in clinical settings [26,27]. To address these limitations, our research group has proposed an innovative therapeutic strategy that synergizes the effects of taxifolin with a temperature-sensitive hydrogel. In our initial investigations, we developed a taxifolin-loaded temperature-sensitive hydrogel drug delivery system based on a polymer matrix of N-isopropylacrylamide, designed specifically for intranasal administration. This system demonstrates a core advantage: it effectively traverses the blood–brain barrier, facilitating targeted delivery of pharmacological agents to the central nervous system. This not only enhances the bioavailability and biocompatibility of taxifolin but also overcomes the limitations inherent in traditional drug administration methods. Consequently, this approach enables potent and effective therapeutic outcomes in the brain while minimizing systemic exposure. Furthermore, preliminary findings suggest that taxifolin may possess the capacity to modulate mitochondrial function and mitigate oxidative damage. Nevertheless, the precise neuroprotective mechanisms underlying these effects remain elusive. Building on our previous work, this study aims to further elucidate the mechanisms by which taxifolin facilitates improvements in AD through comprehensive in vivo experiments.

Based on the aforementioned research background and the preceding findings of our research group, this study employs a research strategy that integrates network pharmacology with molecular docking to construct a comprehensive interaction network elucidating the effects of taxifolin in targeting AD. We systematically screen for core targets associated with the anti-AD effects of taxifolin and further speculate on the key regulatory signaling pathways involved. Utilizing an Okada acid (OA)-induced AD model, we systematically investigate the neuroprotective mechanisms of a thermosensitive hydrogel formulation of taxifolin intended for intranasal administration. This approach facilitates the identification of specific molecular targets and establishes a theoretical foundation for developing a combinatorial treatment strategy to address the detrimental cycle between ferroptosis and mitochondrial dysfunction in AD.

## 2. Materials and Methods

### 2.1. Materials

Taxifolin, purity > 98.0%, N-isopropylacrylamide (NIPAM), tetramethylethylenediamine (TEMED), potassium persulfate (KPS), and polyvinylpyrrolidone (PVP-K30) were all purchased from Shanghai McLean (Shanghai, China); methacrylated gelatin (GelMA, grafting rate of 90%) was purchased from Wenzhou Shuhe Biotechnology Co., Ltd. (Wenzhou, China); deionized water was purchased from Solaibao (Beijing, China); an ELISA kit and mitochondrial complex I kit were purchased from Shanghai Enzyme linked Biotechnology Co., Ltd. (Shanghai, China); WB-related antibodies were purchased from Beijing BioSense Biotechnology Co., Ltd. (Beijing, China); and all other reagents were analytical-grade reagents available from commercial sources.

### 2.2. Preparation of Temperature-Sensitive Hydrogel Loaded with Taxifolin (TF-Gel)

In the preparation of the taxifolin hydrogel, powdered N-isopropylacrylamide (NIPAM, 1.02 g) was dissolved in deionized water (3 mL) to create a homogenous solution. Following this, 0.5 wt% methacryloyl gelatin (GelMA, 1.5 mL) was added, and the mixture was stirred continuously for 20 min to ensure complete integration. Subsequently, a solution of 2 wt% potassium persulfate (KPS, 1 mL) and tetramethylethylenediamine (TEMED, 1 mL) was added sequentially. Thereafter, the resulting mixture was transferred to an ice water bath and stirred for an additional 10 min to achieve a uniform precursor solution. To prepare the drug solution, taxifolin (4.0 mg) and polyvinylpyrrolidone K30 (PVP-K30, 0.6 mg) were accurately weighed and dissolved together in deionized water (1.0 mL). The mixture was thoroughly stirred to ensure homogeneity. Subsequently, 1.0 mL of the aforementioned PNIPAM precursor solution was combined with the drug solution under continuous stirring, resulting in the formation of a taxifolin hydrogel with a final concentration of 0.4% (*w*/*v*) of taxifolin. To promote full swelling, all prepared hydrogels were stored at 4 °C for 24 h. To eliminate any entrained air bubbles, the resulting taxifolin hydrogel was then centrifuged at 20 °C at 12,000 r/min for 10 min before being set aside for further applications. The physicochemical characterization, stability, and in vivo safety profile of this formulation have been systematically established in our prior study [28].

### 2.3. Prediction of Target Genes for Taxifolin

Retrieve the structural information of taxifolin and its Canonical SMILES from the PubChem database, import the Canonical SMILES into the SuperPred database for target prediction, and supplement the target data with the TCMSP database. Merge and deduplicate the targets from both databases to determine the drug target.

### 2.4. Alzheimer’s Disease Target Prediction

A systematic search was conducted using the keyword “Alzheimer’s disease” in the GeneCards database and OMIM database to identify potential targets related to Alzheimer’s disease, integrate and deduplicate the obtained disease targets, and retain and identify Alzheimer’s disease targets.

### 2.5. Prediction of Potential Targets of Taxifolin and AD

Using Cytoscape (3.8.2) software, intersection mapping of taxifolin targets and AD targets was performed to obtain the intersection genes. Then, a “taxifolin AD target” network was constructed and a Venn diagram was drawn to analyze the interaction between taxifolin and AD.

### 2.6. Construction of Taxifolin Target Regulatory Network

Using Cytoscape (3.8.2) software, the obtained intersection targets were imported, and taxifolin and its target were used as network nodes. The interaction relationship between the two was used as an edge to construct a “taxifolin target” network diagram, aiming to reveal the mechanism of taxifolin’s anti-Alzheimer’s disease effect.

### 2.7. Construction of Target Protein Interaction Network (PPI)

Upload the taxifolin AD intersection gene to the interaction database string to construct a target protein interaction network (PPI), set the species as “Homo sapiens”, set the minimum required interaction score to 0.7, and maintain the default settings for other parameters. The degree size is based on the node size and color response, with larger nodes indicating a higher degree value. The combination score is determined by the thickness of the lines, with thicker lines indicating a larger combination score. The analysis results screen out core targets to construct a PPI network diagram.

### 2.8. Gene Ontology (GO) Function and Pathway Enrichment Analysis

The R language integrated development environment Rstudio software (Rstudio 2023.12.1.0) was used to perform Gene Ontology Analysis (GO) and Kyoto Encyclopedia of Genes and Genomes (KEGG) enrichment analysis on the screened targets. Using *p* < 0.05 as the standard for enrichment screening, the top ten enrichment results were selected from three aspects: biological process (BP), cellular component (CC), and molecular function (MF). To elucidate the target signaling pathway of taxifolin in the treatment of Alzheimer’s disease, KEGG pathway enrichment analysis was performed, and the top 20 signaling pathway maps were drawn using R (Rstudio 2023.12.1.0).

### 2.9. Molecular Docking

Generally speaking, in the protein–protein interaction network, the degree value of proteins is positively correlated with their importance in disease treatment, that is, proteins with higher degree values are more likely to play an important role in the treatment of Alzheimer’s disease with taxifolin. Therefore, molecular docking software AutoDock Vina (1.1.2) was used to perform molecular docking between taxifolin and key targets to verify their interaction activity. PyMOL 2.5.5 and LigPlot 1.4.2 were used for interaction mode analysis and visualization. Core targets for molecular docking were selected based on the following criteria: (1) degree centrality in the PPI network; (2) enrichment in AD-related KEGG pathways; (3) relevance to ferroptosis and neuroinflammation; (4) binding energy ≤ −5 kcal/mol from molecular docking.

### 2.10. Induction and Grouping of AD Mice

In this study, 90 male ICR mice, weighing 20 ± 2 g, were purchased from Changchun Yisi Experimental Animal Technology Co., Ltd. (Changchun, China) (certificate number: SCXK-2020-0001). After one week of adaptive feeding in a standard laboratory environment (22 ± 2 °C, 60 ± 5% humidity, 12 h of alternating day–night), the mice were randomly divided into six groups: control group (Control), AD model group (AD), nasal injection of high-dose TF-Gel plus ferroptosis inducer Erastin group (H-IN+Erastin), positive control group (H-IG) administered with taxifolin suspension by gavage, nasal injection of low-dose TF-Gel group (L-IN), and nasal injection of high-dose TF-Gel group (H-IN). Based on the core exploration indicators of behavioral science, ferroptosis, and mitochondrial dysfunction, the required sample size for the experiment was calculated. We set n = 10 to ensure sufficient power.

Mice were anesthetized by intraperitoneal injection of 0.2 mL/20 g of 0.4% pentobarbital sodium, fixed on a stereotaxic device, and exposed to the skull. The bilateral hippocampal CA1 region was targeted for injection using the following stereotaxic coordinates relative to bregma: anteroposterior (AP) = −2.0 mm, mediolateral (ML) = ±1.5 mm, and dorsoventral (DV) = −1.5 mm. An AD mouse model was induced by injection of 2 μL of Okada acid (10 μg/0.1 mL) (parameters: injury depth of 2.5 mm, injection needle entry rate into the brain of 1 mm/min, injection rate of 0.2 μL/min, and residence time of 1 min). The blank group was injected with 2 μL of physiological saline instead of Okada acid. After stopping the injection, the wound was sutured and penicillin was injected intraperitoneally for 3 consecutive days to prevent infection. The mice were placed in a constant temperature incubator at 25 °C to ensure a constant body temperature.

After 3 days of postoperative observation of the physiological status of mice, it was confirmed that all mice had returned to normal. Different administration methods and drug formulations were used to administer drugs to AD mice in each treatment group. Based on the preliminary experimental screening of the research group, the H-IG group of mice was determined as the positive control group. The mice in this group were given taxifolin suspension aqueous solution (40 mg/kg/d, 0.5% carboxymethyl cellulose sodium suspension) by gavage. Based on the previous pharmacokinetic and bioavailability equivalent conversion, the dosage of the H-IN group was 2 mg/kg, and the dosage of the L-IN group was 1 mg/kg, aiming to verify their high efficiency at low doses. In addition, to elucidate the importance of ferroptosis in the improvement of AD pathology by TF-Gel, an intervention group was added to the experiment in combination with ferroptosis inducer Erastin on the basis of TF-Gel treatment. Except for the model group, all other groups of mice were continuously administered to for 60 days. The exclusion criteria mainly included the following situations: postoperative death or serious health deterioration unrelated to treatment; technical malfunctions occurring during drug administration or sample collection; and not meeting the criteria for disease model assessment (such as behavioral experiments not meeting the standards, etc.). The daily administration time was 10 am, and the medication was administered in the order of grouping, with no change in the administering personnel. Animals were only included in the study if they completed the entire experimental process without any complications.

Blinding was not implemented in this study. All personnel involved in animal allocation, experimental procedures, outcome assessment, and data analysis were aware of the group identities.

### 2.11. Morris Behavioral Experiment

Five days before the end of treatment, a Morris water maze (MWM) and computer video tracking system were used to measure, record, and evaluate the neural memory, cognition, and motor function of mice. Before formally recording the experimental results, mice needed to be trained continuously for 5 days, with each mouse alternating 5 times a day. The escape latency for each mouse was calculated as the mean of the four training trials per day (excluding the first trial as adaptation), and these daily means were used for statistical analysis. After the training, behavioral video analysis software v2.0 was used to conduct localization and navigation experiments on mice for 180 s. We used the time it took for mice to find an underwater platform as the evaluation criterion. To minimize potential bias, behavioral tests were conducted by two independent experimenters who were blinded to the group allocations, and the results from both experimenters were averaged for statistical analysis.

### 2.12. Biochemical Parameter Evaluation

#### 2.12.1. Evaluation of Serum Biochemical Parameters

After behavioral testing, blood samples were collected from mice using the mandibular venous plexus blood collection method. The collected blood samples were placed in anticoagulant tubes soaked in heparin sodium and allowed to stand at room temperature for 10 min. Then, they were placed in a centrifuge with the temperature set at 20 °C and a speed of 3600 r/min for 15 min. The supernatant was collected and placed at −80 °C for testing. According to the instructions of the ELISA kit manufacturer, we detected the levels of oxidative stress indicators reactive oxygen species (ROS), superoxide dismutase (SOD), as well as pro-inflammatory factors interleukin-1β (IL-1β), interleukin-6 (IL-6), and tumor necrosis factor-alpha (TNF-α) expression.

#### 2.12.2. Evaluation of Biochemical Parameters of Hippocampal Tissue

After the blood sample collection was completed, the mice were euthanized using the cervical dislocation method. Then, the mice were placed on an ice table and quickly dissected, brain tissue was removed, and the hippocampus was separated. Using a mass to volume ratio of 1:9 as the standard, the hippocampus was immersed in sterile physiological saline to prepare a 10% hippocampal homogenate solution. According to the manufacturer’s instructions for the ELISA kit, the levels of oxidative stress markers malondialdehyde (MDA), nicotinamide adenine dinucleotide phosphate (NADPH) oxidase activity, antioxidant enzyme glutathione peroxidase (GSH-Px), neurotrophic factors (brain-derived neurotrophic factor (BDNF) and transforming growth factor-beta (TGF-β)), neurotoxic factor glutamate glutamic acid (Glu) and its downstream indicator lactate dehydrogenase (LDH), mitochondrial energy, and functional core factor adenosine triphosphate (ATP) were measured. According to the manufacturer’s instructions for the mitochondrial complex I activity assay kit, we detected the expression of mitochondrial complex I activity.

### 2.13. Histopathological Examination

#### 2.13.1. H&E Staining

Wash the hippocampal tissue with PBS, fix it in a 4% paraformaldehyde solution, dehydrate and fix the sample, embed it in paraffin, deparaffinize it with xylene for 10 min, dehydrate it with gradient concentration ethanol (100%, 95%, 90%, 80%), slice it with a thickness of 5 μm, and seal it with neutral gum. All sections were stained with H&E staining solution and observed under an Olympus BX51 optical microscope (Olympus Corporation, Tokyo, Japan). Images of the hippocampal CA1, CA3, and DG regions were collected for histopathological analysis. All histological sections were coded and analyzed by a researcher unaware of the treatment group assignments to ensure unbiased assessment.

#### 2.13.2. Nissl Stainin

Freeze a section of the paraffin sample at −20 °C with a thickness of 5 μm. All sections were stained with toluidine blue and observed in real time under an optical microscope. After washing with PBS, they were immersed in 95% ethanol for differentiation, dehydrated in 100% ethanol, transparent in xylene for 10 min, and sealed with neutral gum. Finally, the slices were observed under an Olympus BX51 optical microscope, and images of the hippocampal CA1, CA3, and DG regions were collected for histopathological analysis. Image Pro Plus 6.0 software was used to analyze the average optical density (AOD) of the stained slices.

### 2.14. Immunofluorescence Staining Detection

The expression of ROS content in the CA1, CA3, and DG regions of mouse hippocampi was detected using immunofluorescence staining. The prepared frozen sections were incubated with 50 μM DHE for 1 h under completely dark and room-temperature conditions and washed with PBS to remove unbound probes. Observe the slices under an Olympus BX51 optical microscope, collect fluorescence images of the hippocampal CA1, CA3, and DG regions, and analyze the average optical density (AOD) of the stained slices using Image Pro Plus 6.0 software.

### 2.15. TEM Microstructure Detection

#### 2.15.1. Detection of Mitochondrial Ultrastructure Morphology

Take fresh mouse hippocampus tissue and quickly cut it into 1 mm^3^ cubes in cold PBS. Fix it at 4 °C using a mixture of 2.5% glutaraldehyde and 2% paraformaldehyde (prepared with 0.1 M PBS phosphate buffer), soak overnight, rinse with PBS for 15 min, and fix it in a mixture of 1% osmium tetroxide and 0.8% potassium ferrocyanide at 4 °C for 2 h. Dehydrate the sample in a 50% to 100% ethanol gradient, fix it in acetone, embed it in Spurr resin, and polymerize it at 70 °C for 24 h. Use a Leica EM UC7 microtome (Wetzlar Germany) to cut the sample into 70 nm ultra-thin slices, place them on a copper mesh, immerse them in hematoxylin solution and lead citrate solution for staining, and place them under a TEM transmission electron microscope (FEI Talos, Hillsboro, OR, USA). Observe mitochondrial morphology under L120C and collect images, followed by analysis using Image Pro Plus 6.0 software.

#### 2.15.2. Identification of Morphological Characteristics of Ferroptosis

TEM transmission electron microscopy was used to observe the morphological characteristics of ferroptosis in hippocampal neurons, including typical features such as mitochondrial volume, membrane density, mitochondrial spine, and outer mitochondrial membrane morphology. The morphology of the cytoplasmic membrane and nucleus was also observed to distinguish it from cell necrosis and apoptosis.

### 2.16. JC-1 Probe for Detecting Mitochondrial Membrane Potential (MMP)

#### 2.16.1. Flow Cytometry Analysis

Using the JC-1 mitochondrial detection kit (Beyotime, Shanghai, China) and flow cytometry, MMP was examined. Fresh mouse hippocampal tissue was rapidly placed in pre-cooled neuronal dissociation buffer (containing papain) and incubated at 37 °C for 15 min to prepare a single-cell suspension. Cells were resuspended in culture medium containing 5 μM JC-1 probe and incubated at 37 °C for 30 min in the dark. After washing twice with ice-cold PBS, the suspension was resuspended in PBS and placed on ice, followed by analysis using flow cytometry. JC-1 fluorescence forms aggregates at high MMP, emitting red fluorescence, and forms monomers at low MMP, emitting green fluorescence. The average ratio of red/green fluorescence intensity in the cell population is calculated to quantitatively evaluate changes in mitochondrial membrane potential.

#### 2.16.2. Fluorescence Microscopy Imaging Analysis

To further observe the spatial distribution of mitochondrial membrane potential in situ, fluorescence microscopy imaging was used for analysis. After fixing fresh mouse hippocampi with 4% paraformaldehyde, they were dehydrated to prepare 20 µm frozen sections. They were incubated with 5 μM JC-1 working solution in the dark at 37 °C for 30 min, gently washed with PBS, and repeated three times. Anti-quenching sealing tablets containing DAPI were used to seal the slices and placed under a laser confocal microscope for observation. In the high-membrane-potential region, the fluorescence signal appears as red dots, and when the membrane potential decreases, it turns into green fluorescence. Use ImageJ software 1.8.0 to analyze the red/green fluorescence intensity ratio of the target area for semi-quantitative evaluation.

### 2.17. Western Blot Analysis

Western blot (WB) was used to detect the pathological expression of synaptic apoptosis pathway-related proteins tau, p-Tau, Synaptophysin (SYP), post synaptic density protein 95 (PSD95), apoptosis-inducing factor (AIF), Bcl-2, Bax, and cleaned-caspase3 in hippocampal tissue, as well as the expression levels of ferroptosis regulatory signaling factors ferroportin 1 (Fpn1), transferrin receptor 1 (TfR1), divalent metal transporter 1 (DMT1), glutathione peroxidase 4 (GPX4), dihydroorotate dehydrogenase (DHODH), and signaling pathway factors toll-like receptor 4 (TLR4), nuclear factor kappa-B (NF-κB), hypoxia-inducible factor 1-alpha (HIF-1α), inducible nitric oxide synthase (iNOS), vascular endothelial growth factor (VEGF), and glucose transporter 1 (Glut1). The relevant proteins in each group of hippocampal tissue were lysed using RIPA lysis buffer to prepare hippocampal homogenate, and the total protein concentration was determined using a BAC protein detection kit (Waltham, MA, USA). The protein was separated using 5% sodium dodecyl sulfate polyacrylamide gel electrophoresis (SDS-PAGE) and then transferred to the polyvinylidene fluoride (PVDF) membrane. Due to the limited loading capacity of each gel, samples from all 10 animals per group were processed across multiple membranes for each target protein. All samples from all membranes were included in the subsequent densitometric analysis and statistical comparisons. Representative blots are shown in the figures, while the quantitative data reflect the complete dataset from all biological replicates (n = 10). At room temperature, 0.1% Tween-20 tris Buffered saline (TBS) was used to seal the membrane with 5% fat-free milk for 2 h. At 4 °C, the membrane was incubated with the primary antibody overnight, then the corresponding secondary antibody was added, and incubated at room temperature for 2 h. β-actin was used as the internal loading control for all Western blot experiments. Protein expression levels were normalized to β-actin and expressed as fold change relative to the control group. ECL reagent was used to display Western blot, and enhanced chemiluminescence (ECL) substrate and KODAK image workstation 4000 MM (Carestream Health, Inc., New Haven, CT, USA) were used to detect the intensity of the blot. Image J was used to quantify the grayscale values. Quantification of Western blot bands was performed by an investigator who was blinded to the experimental groups throughout the analysis process.

### 2.18. Statistical Processing

All data were expressed as mean ± standard deviation (SD). Statistical analyses were performed using GraphPad Prism 8.0.2 (GraphPad Software, Boston, MA, USA) and Origin 2024 (OriginLab, Northampton, MA, USA). Prior to parametric analysis, normality was assessed using the Shapiro–Wilk test, and homogeneity of variance was evaluated using Levene’s test. All datasets met the assumptions of normal distribution and equal variance (*p* > 0.05), justifying the use of parametric tests. For comparisons between two groups, unpaired two-tailed Student’s *t*-test was used. For multiple group comparisons, one-way or two-way analysis of variance (ANOVA) was first performed, followed by the Bonferroni multiple comparison test (S-N-K method) for post hoc analysis. Two-way repeated measures ANOVA was used for behavioral data (escape latency over training days). Post hoc analyses were conducted only when ANOVA indicated significant overall effects (*p* < 0.05). The differences were considered statistically significant when *p* < 0.05, *p* < 0.01, or *p* < 0.001. Otherwise, it is considered to have no statistical significance.

## 3. Results

### 3.1. Network Pharmacology and Molecular Docking Target Analysis

**Figure 1:**
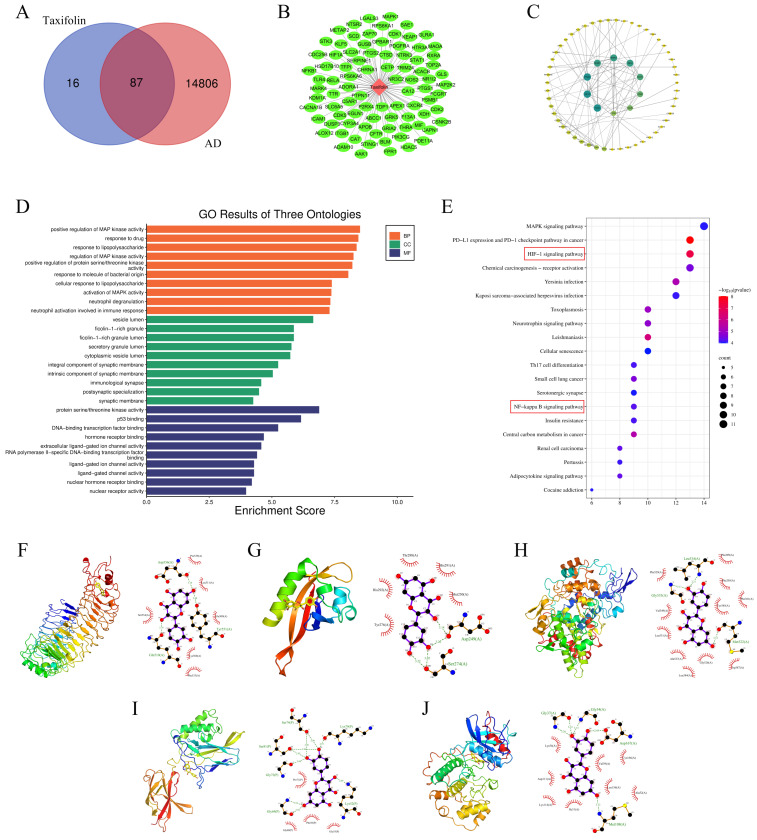
Network pharmacology and molecular docking analysis results of taxifolin–AD. (**A**) Venn diagram of taxifolin and AD intersection targets. (**B**) Taxifolin–target regulatory network diagram. (**C**) PPI network diagram of taxifolin–AD intersection target. (**D**) GO function enrichment analysis. (**E**) KEGG pathway enrichment analysis bubble plot. (**F**–**J**) Analysis of the interaction mode between taxifolin and TLR4, HIF1A, PTGS2, NFKB1, and MAPK1 proteins.

The online platform network database was meticulously screened to identify intersecting and redundant targets between taxifolin and AD. The resulting Venn diagram analysis (Figure 1A) illustrated the existence of 87 intersecting genes between taxifolin and AD targets. Following the intersection analysis, the identified target genes were visualized using Cytoscape software, yielding a multi-target network characterized by 88 nodes and 87 edges, which formed a distinct star-shaped topological structure (Figure 1B). Subsequently, a protein–protein interaction (PPI) network was constructed (Figure 1C), comprising 61 nodes and 94 edges. Utilizing a Cytoscape plugin, we further refined and identified core target genes, resulting in the selection of five pivotal targets: TLR4, HIF1A, PTGS2, NFKB1, and MAPK1. To comprehensively understand the biological implications of these core targets, we conducted Gene Ontology (GO) enrichment analysis (Figure 1D) and Kyoto Encyclopedia of Genes and Genomes (KEGG) enrichment analysis (Figure 1E) on the identified intersection targets associated with taxifolin in the context of AD. These analyses reveal potential pathways and biological processes that could be regulated by the interaction of taxifolin with these core targets, providing insights into its therapeutic potential for AD.

To further elucidate the multi-target anti-AD mechanism of taxifolin, we employed a combination of network pharmacology analysis and molecular docking methodologies. Specifically, we conducted semi-flexible docking studies between taxifolin and key intersecting core targets to investigate the ligand’s binding affinity and interaction stability with each target. As presented in Table 1, the binding energies for taxifolin with five core target proteins are all below −5 kcal/mol, underscoring its capability to access the active sites of these proteins while demonstrating a high degree of affinity and binding stability. Figure 1F–J illustrate the interaction modes between taxifolin and the core target proteins, featuring 3D molecular docking structures and 2D intermolecular interaction diagrams. Detailed analysis of the binding modes revealed that taxifolin forms stable hydrogen bonds with key amino acid residues in the active sites of these proteins. As shown in Figure 1F–J, the hydrogen bond distances ranged from 2.76 to 3.32 Å, all within the typical range of 2.5–3.5 Å for stable interactions (specific distances: TLR4: Tyr 551 2.80 Å, Asp 536 3.14 Å, Gln 510 2.76 Å; HIF1A: Ser 274 3.22/3.02 Å, Asp 249 3.20/3.14 Å; PTGS2: Met 522 3.12 Å, Leu 534 3.21 Å, Gly 533 3.11 Å; NFKB1: Lys 52 2.89 Å, Lys 79 2.81 Å, Ser 74 2.90 Å, Ser 81 2.86 Å, Gly 72 2.96 Å, Gly 69 2.95–3.32 Å; MAPK1: Met 108 3.12 Å, Asp 167 2.95 Å, Gly 34 3.13 Å, Gly 37 3.12/3.26 Å). The findings reveal that taxifolin can form stable and specific networks with the active sites of various core target proteins. Notably, the active sites of TLR4, NFKB1, and HIF1A exhibit relatively stronger binding affinities and more stable structural interactions with taxifolin, suggesting their potential significance in the compound’s therapeutic action against AD.

### 3.2. TF-Gel Improves Behavioral Cognitive Impairment in AD Mice

#### 3.2.1. Morris Water Maze Thermal Imaging Trajectory Map

**Figure 2:**
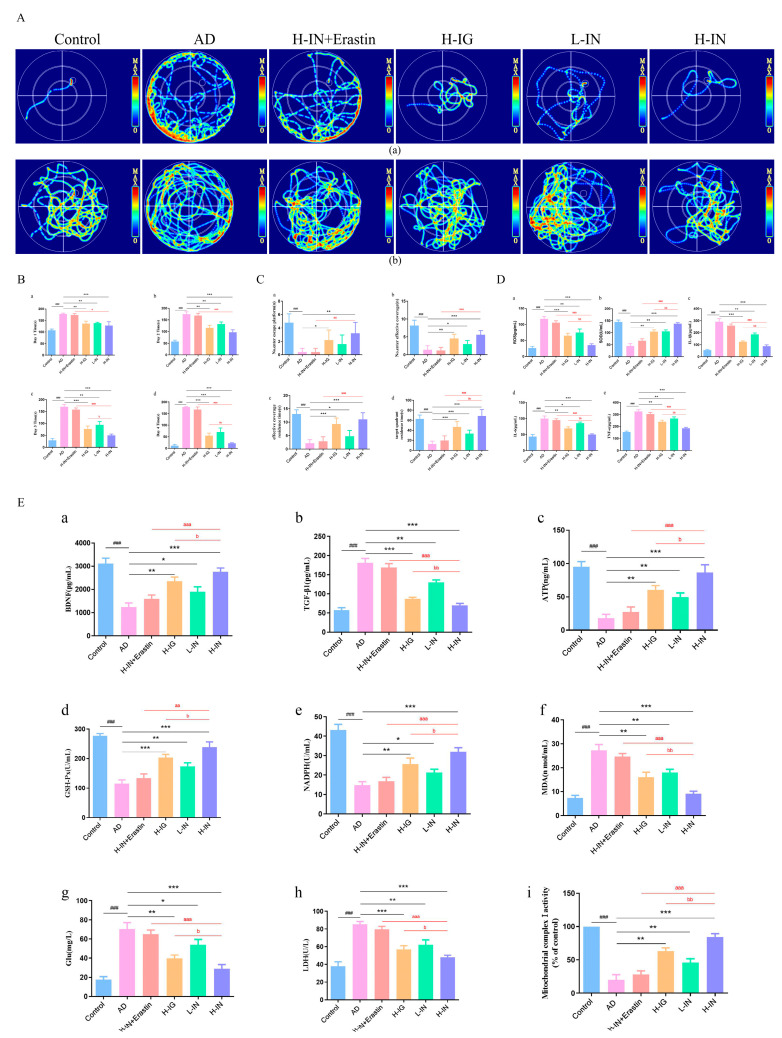
The effect of TF-Gel on behavioral cognitive impairment and related biochemical indicators in AD mice. (**A**) Thermal imaging trajectory in the Morris water maze positioning navigation experiment and spatial probe test. (**a**) shows the activity trajectories of positioning navigation experiment, and (**b**) shows the activity trajectories of spatial probe test. (**B**) Quantitative analysis of positioning navigation experiment from day 1 to day 4. Positioning navigation experiment in the Morris water maze. (**a**) Day 1 time. (**b**) Day 2 time. (**c**) Day 3 time. (**d**) Day 4 time. (**C**) Quantitative analysis of spatial probe test. Spatial probe test in the Morris water maze. (**a**) No.enter escape platform. (**b**) No.enter effective coverage. (**c**) effective coverage residence time. (**d**) target quadrant residence time. (**D**) Evaluation of biochemical parameters ROS (**a**), SOD (**b**), IL-1β (**c**), IL-6 (**d**) and TNF-α (**e**) in serum. (**E**) Evaluation of biochemical parameters BDNF (**a**), TGF-β (**b**), ATP (**c**), GSH-Px (**d**), NADPH (**e**), MDA (**f**), Glu (**g**), LDH (**h**) and Mitochondrial complex I activity (% of control, (**i**)) in hippocampus. ^###^ *p* < 0.001 vs. control group; * *p* < 0.05, ** *p* < 0.01, *** *p* < 0.001 vs. AD group; ^a^ *p* < 0.05, ^aa^ *p* < 0.01, ^aaa^ *p* < 0.001 vs. H-IN+Erastin group; ^b^ *p* < 0.05, ^bb^ *p* < 0.01 vs. H-IG group ($\bar{x}$ ± s, n = 10).

Utilizing video tracking technology, we recorded the activity trajectories of mice across the various experimental groups during the final day of the positioning navigation experiment, as well as the spatial probe test. From these recordings, we generated thermal infrared trajectory maps and selected representative examples from each group for analysis (Figure 2A). As illustrated in Figure 2(Aa), during the positioning and navigation experiment, mice in the control group swiftly located the underwater escape platform, which was strategically placed at the center of the first quadrant of the pool, within a remarkably short duration training. In contrast, the activity trajectories of the AD group mice exhibited aimless and erratic movement, predominantly along the periphery of the pool. Upon intervention, all treated groups of mice demonstrated the ability to discover the underwater escape platform within a specified timeframe. Notably, the H-IN group exhibited the most significant improvement in performance. However, this beneficial effect was notably diminished upon administration of the ferroptosis inducer, Erastin. These findings underscore the influence of treatment on navigational capabilities in the context of AD-induced impairments.

In the space exploration experiment (Figure 2(Ab)), the activity trajectories recorded for each group of mice over the designated time mirrored the trends observed in the positioning and navigation experiment. Mice in the control group predominantly occupied areas near the target quadrant, indicating a clear retention of spatial memory regarding the location of the target platform. Conversely, mice in the AD group continued to display aimless and irregular movement patterns. The activity ranges of mice in the treatment groups exhibited varying degrees of convergence, demonstrating significantly more purposeful behavior compared to the model group. Among the treatment cohorts, the H-IN group showed a notably better aggregation pattern within the target quadrant than the other treatment groups. Consistently, the administration of the ferroptosis inducer Erastin notably reversed the positive effects observed in the H-IN group. These results emphasize the therapeutic potential of the treatments in enhancing spatial awareness and navigational abilities in the context of AD.

#### 3.2.2. Localization Navigation Experiments

The localization navigation experiments assessed the time taken for each group of mice to locate the underwater escape platform from day 1 to day 4, as illustrated in Figure 2B. Compared to the control group, the escape latency in the AD group was significantly prolonged. When assessed against the model group, the escape latency in each treatment group exhibited a notable reduction. Post hoc multiple comparison analyses revealed that the H-IN group demonstrated the most significant decrease in escape latency, surpassing the effect observed in the H-IG group. Furthermore, when compared to the H-IN+Erastin group, the improvement observed in the H-IN group was also significant.

#### 3.2.3. Spatial Probe Test

The number of times each group of mice entered the escape platform zone, entered the target quadrant, remained within the escape platform zone, and stayed in the target quadrant over a specified period were measured separately. The experimental results are presented in Figure 2C. Compared to the control group, all four measurement indicators in the AD group were significantly reduced. In contrast, each treatment group exhibited a significant improvement in both the quantity and duration of these indicators when compared to the AD group. Inter-group analysis revealed that the H-IN group demonstrated the most pronounced improvement. Furthermore, the beneficial effects of the H-IN group were significantly diminished by the administration of Erastin. These results collectively suggest that the therapeutic mechanism of TF-Gel in AD may be associated with ferroptosis.

### 3.3. Biochemical Parameter Evaluation

As illustrated in Figure 2D,E, the AD group exhibited significant oxidative stress when compared to the blank group. Notably, serum ROS levels in this group were markedly elevated, while SOD levels significantly declined. Additionally, MDA levels in the hippocampus showed a significant increase, and the expression of NADPH oxidase and the antioxidant enzyme GSH-Px significantly decreased. Concurrently, the AD group demonstrated pronounced inflammatory responses, as evidenced by significant increases in serum levels of pro-inflammatory cytokines IL-1β, IL-6, and TNF-α. Furthermore, in terms of neurological expression, the AD mice exhibited a dual disturbance in the neural nutrition regulatory system alongside increased excitotoxicity. BDNF levels in their hippocampal tissue were significantly reduced, while levels of TGF-β, Glu, and their downstream indicator LDH displayed significant increases. ATP content was significantly decreased, while mitochondrial LDH levels were notably elevated in the AD group, while the activity of complex I was significantly diminished, indicating substantial mitochondrial dysfunction in the AD group. Relative to the AD group, each treatment group ameliorated the abnormal expression of the aforementioned biochemical indicators, with the H-IN group demonstrating the most pronounced reversal effect, significantly surpassing that of the H-IG group. The administration of Erastin notably inhibited all observed improvements, suggesting that the beneficial effects of the H-IN group are mediated by ferroptosis.

### 3.4. Histopathological Analysis

#### 3.4.1. H&E Staining

**Figure 3:**
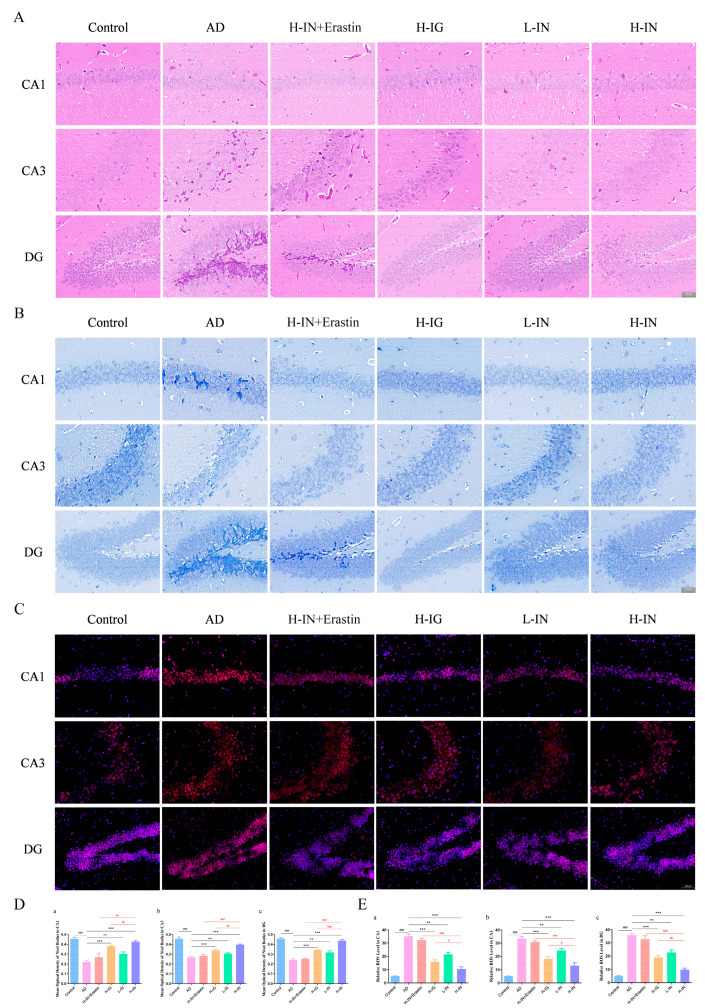
Histopathology, neuronal activity, and oxidative stress assessment of hippocampal CA1, CA3, and DG regions in AD mice by TF-Gel. (**A**) Representative images of H & E staining in the hippocampus subregions. (**B**) Representative images of neuronal Nissl staining in the hippocampus subregions. (**C**) Representative immunofluorescence images of ROS in the hippocampus subregions. (**D**) Hippocampal subregion CA1 (**a**), CA3 (**b**), DG (**c**) with Nissl staining AOD. (**E**) Hippocampal subregion CA1 (**a**), CA3 (**b**), DG (**c**) with ROS immunofluorescence staining AOD. Scale bar = 100 μm (applies to all panels). ^###^ *p* < 0.001 vs. control group; ** *p* < 0.01, *** *p* < 0.001 vs. AD group; ^aa^ *p* < 0.01, ^aaa^ *p* < 0.001 vs. H-IN+Erastin group; ^b^ *p* < 0.05, ^bb^ *p* < 0.01, ^bbb^ *p* < 0.001 vs. H-IG group ($\bar{x}$ ± s, n = 5).

The H&E staining results (Figure 3A) showed that the hippocampal neurons in the blank group of mice were sorted neatly, with distinct layers, clear structures, and full cell bodies, while the hippocampal neurons in the OA-induced AD group of mice were loosely sorted, disordered in hierarchy, wrinkled in cell bodies, and decreased in quantity, indicating that OA caused significant damage to hippocampal neurons. Compared with the AD group, the degree of neuronal damage was significantly reduced in all treatment groups, with the H-IN group showing a better neuronal performance, especially in the CA1 and DG regions. However, the H-IN group neurons with added Erastin still showed significant pathological damage.

#### 3.4.2. Nissl Staining

Under an optical microscope, stained cells can be observed to be blue–purple in color, as shown in Figure 3B. The hippocampal neurons in the control group are arranged in a regular and dense manner. Compared with the blank group, the hippocampal neurons in the AD group show a significantly sparse and disordered order, with larger intercellular spaces, reduced numbers, and a dissolved state. After treatment, the hippocampal neuron status of each treatment group has been significantly improved, with the H-IN group showing the most significant improvement effect. The expression of AOD in each group further confirmed the above results, as shown in Figure 3D. The higher the AOD value, the stronger the activity of Nissl bodies in this region. Compared with the blank group, the AOD values in the CA1, CA3, and DG regions of the hippocampus in the model group were significantly reduced. Compared with the model group, the AOD values in all three regions of the treatment group were significantly increased to varying degrees. Among them, the AOD value increase effect in the H-IN group was the most significant, and the improvement effect was stronger than that in the H-IG group, especially in the CA1 and DG regions. This may be related to the intranasal administration method of the H-IN group. Meanwhile, mice treated with both H-IN and Erastin showed similar neuronal performances and AOD value expression to the AD group. These results further demonstrate from a morphological perspective that taxifolin may be associated with the ferroptosis injury pathway in improving the AD process.

### 3.5. Immunofluorescence Staining Analysis

The expression of ROS activity demonstrates a positive correlation with immunofluorescence intensity, as illustrated in Figure 3C,E. In comparison to the control group, the ROS fluorescence intensity observed in the hippocampal CA1, CA3, and DG neurons of the AD group mice was significantly elevated, indicative of widespread oxidative damage. Following drug treatment, the ROS signals in the hippocampal regions of the H-IN group exhibited pronounced attenuation, while those in the H-IG group displayed a lesser degree of reduction compared to the H-IN group. Notably, the ROS fluorescence intensity in the H-IN+Erastin group was significantly higher than that in the H-IN group, further underscoring the role of ferroptosis as a critical contributor to neuronal oxidative damage.

### 3.6. TEM Microstructure Detection

#### 3.6.1. Mitochondrial Ultrastructure Morphology Detection Results

**Figure 4:**
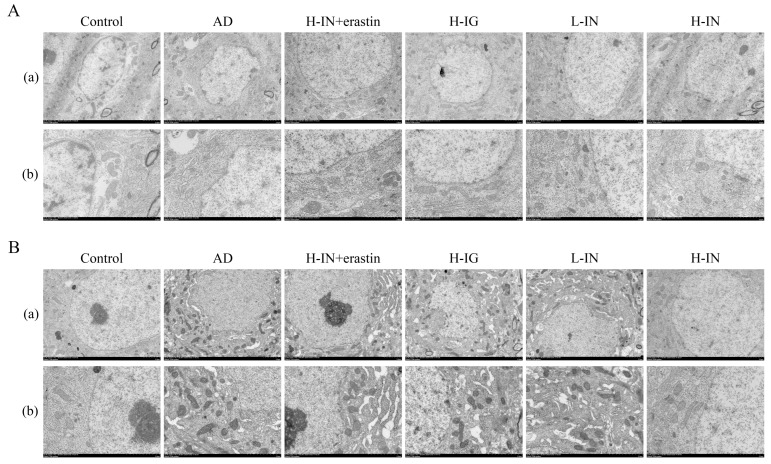
Transmission electron microscopy observation of the effect of TF-Gel on the ultrastructure and ferroptosis-related morphology of hippocampal mitochondria in AD. (**A**) TEM image of mitochondrial ultrastructure. (**B**) TEM image of ferroptosis structure characteristics. (**a**) Magnification: ×2.5 k. (**b**) Magnification: ×5.0 k.

TEM observations revealed that the morphology of mitochondria in the cytoplasm of hippocampal neurons from the control group appeared elliptical, characterized by neatly arranged cristae and clear structural integrity (Figure 4A). In contrast, in the AD group, a considerable number of damaged mitochondrial fragments were observed within the hippocampus. These mitochondria exhibited an altered morphology, disorganized cristae arrangement, and phenomena such as rupture and dissolution, reflecting severe ultrastructural pathological changes. Following treatment across the various groups, there was a notable improvement in mitochondrial morphology, as well as the structural integrity and arrangement of cristae.

#### 3.6.2. Identification Results of Ferroptosis Structure Characteristics

Under high-power transmission electron microscopy, we further observed characteristic structures indicative of the ferroptosis process in neurons from the AD group (Figure 4B). These mitochondria exhibited a decrease or complete loss of cristae, alongside compromised integrity of the outer membrane, while the electron density of the mitochondrial membrane demonstrated an abnormal increase. Following intervention with taxifolin, the overall morphology of mitochondria in the treated group showed significant restoration. Notably, swelling was reduced, the number and clarity of cristae structures were markedly improved, and their arrangement became more orderly. More importantly, characteristic features associated with ferroptosis, such as outer membrane rupture and abnormal increases in membrane density, were significantly diminished in the treatment group. This finding suggests that taxifolin effectively mitigated mitochondrial iron accumulation induced by oxidative agents. The most pronounced improvement was observed in the H-IN group. However, this beneficial effect was notably counteracted by Erastin.

### 3.7. JC-1 Probe for Detecting Mitochondrial Membrane Potential (MMP)

#### 3.7.1. Streaming Graph

**Figure 5:**
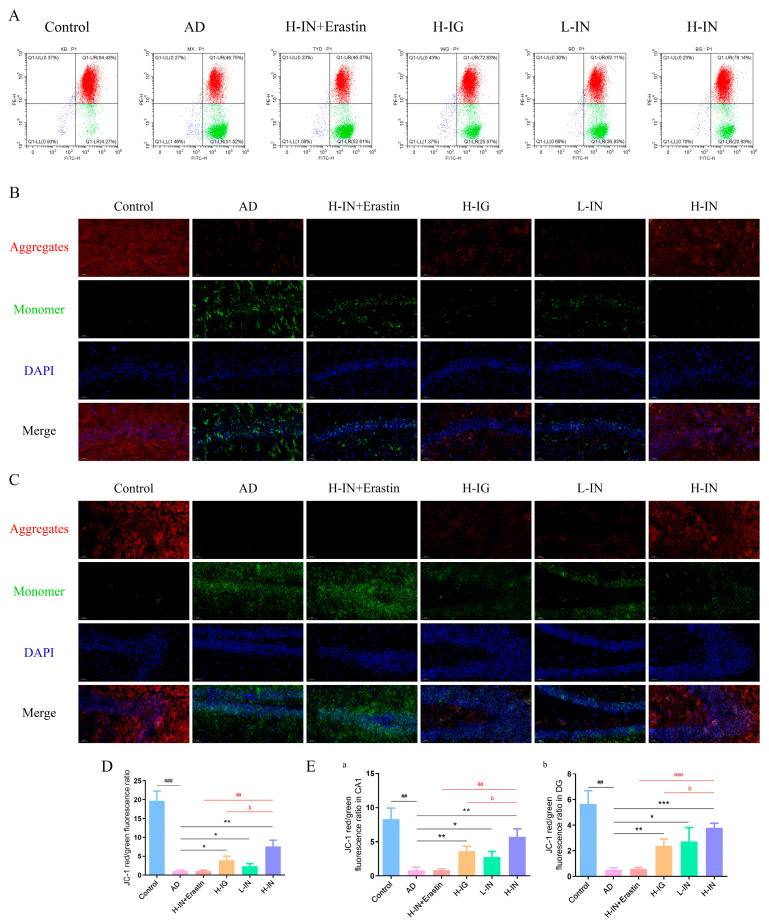
JC-1 detection of the effect of TF-Gel on mitochondrial membrane potential in AD hippocampus. (**A**) JC-1 detection of mitochondrial membrane potential flow cytometry. (**B**) Typical fluorescence image of MMP (JC-1 probe) in hippocampal CA1 region. (**C**) Typical fluorescence image of MMP (JC-1 probe) in hippocampal DG region. (**D**) The ratio of red/green fluorescence in different groups. (**E**) Red/green fluorescence ratio of mitochondrial membrane potential in specific regions of hippocampal subregion CA1 (**a**) and DG (**b**). Scale bar = 50 μm (applies to all panels). ^##^ *p* < 0.01, ^###^ *p* < 0.001 vs. control group; * *p* < 0.05, ** *p* < 0.01, *** *p* < 0.001 vs. AD group; ^aa^ *p* < 0.01, ^aaa^ *p* < 0.001 vs. H-IN+Erastin group; ^b^ *p* < 0.05 vs. H-IG group ($\bar{x}$ ± s, n = 5).

Mitochondrial function is typically assessed by measuring the MMP, which can be quantitatively analyzed using the JC-1 fluorescent probe in conjunction with flow cytometry. The red/green fluorescence intensity ratio (red/green) serves as an indicator of mitochondrial membrane potential, with MMP levels being directly proportional to this ratio. As depicted in Figure 5A,D, the red/green fluorescence intensity ratio in the AD group was significantly reduced compared to the control group, indicating a severe depolarization of the mitochondrial membrane potential. Following treatment, the fluorescence intensity ratio in the treatment group exhibited a significant increase, demonstrating that the intervention effectively restored the mitochondrial membrane potential and stabilized the electrochemical gradient. Notably, the H-IN group exhibited the highest MMP value among all examined groups.

#### 3.7.2. Fluorescence Microscopy Imaging Image

Based on previous histological examinations using hematoxylin and H & E staining and Nissl staining, the findings indicated that the CA1 and DG regions exhibited more pronounced pathological improvements and neuroprotective effects compared to the CA3 region. This observation suggests that the CA1 and DG regions may possess a heightened sensitivity to intranasal delivery of brain-targeted therapies. In pursuit of further validation regarding mitochondrial function enhancement through TF-Gel, this study employed the JC-1 probe to assess mitochondrial membrane potential, focusing specifically on the CA1 and DG regions due to their pronounced responsiveness to therapeutic interventions.

Fluorescence microscopy data for the CA1 region (Figure 5B) and the DG region (Figure 5C) corroborate the conclusions derived from flow cytometry analyses. In the control group, mitochondria predominantly formed bright red JC-1 polymer fluorescent aggregates, indicative of high membrane potential. In contrast, the AD group displayed a significant increase in green JC-1 monomer fluorescence, which was diffusely distributed throughout the cytoplasm, signifying low membrane potential. Additionally, there was a marked reduction in red fluorescence intensity in this group. Following treatment, a noticeable restoration of red fluorescence intensity was observed, accompanied by a corresponding decrease in green fluorescence, thereby indicating an effective improvement in the depolarization state of mitochondrial membrane potential. Among the treated groups, the H-IN group exhibited the most significant recovery of red fluorescence intensity, a finding that was quantitatively substantiated through red-to-green fluorescence ratio analysis (Figure 5E).

### 3.8. Western Blot Analysis

#### 3.8.1. Synaptic Apoptotic Pathway Protein

**Figure 6:**
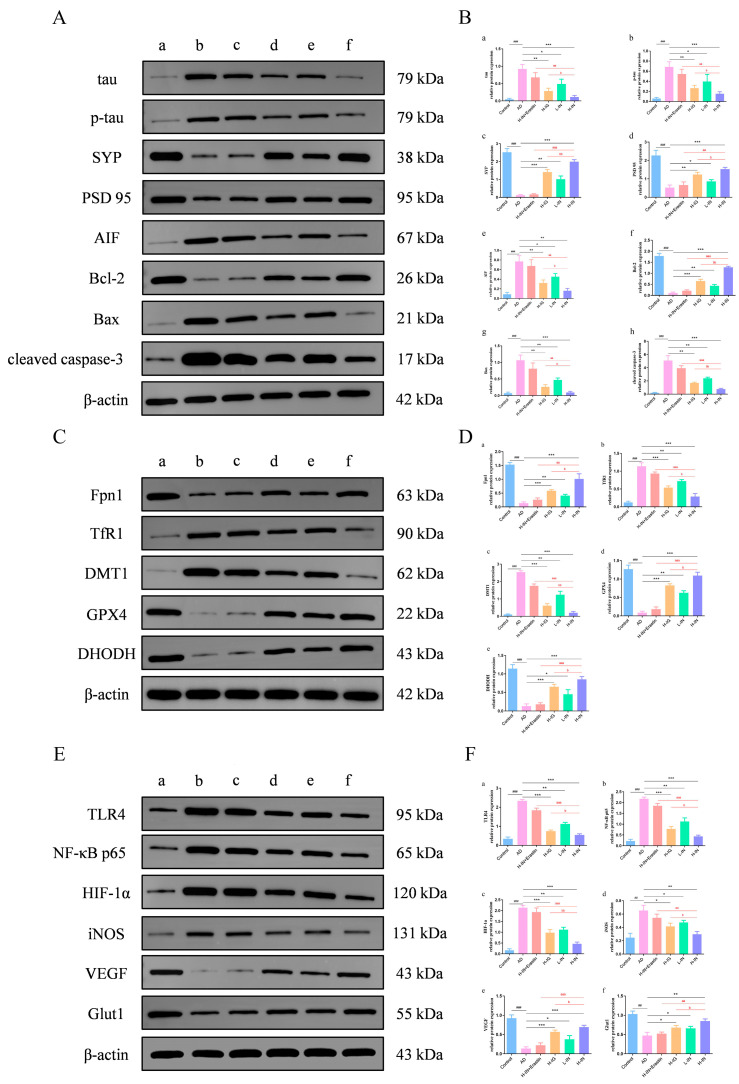
TF-Gel improves AD ferroptosis and related synaptic damage and cell apoptosis through the TLR4/NF-κB/HIF-1α signaling pathway, as validated by WB. (**A**) The protein expression images of tau, p-tau, SYP, PSD95, AIF, Bcl-2, Bax, and cleaved caspase-3 in the hippocampus. (**B**) The protein expression level of tau (**a**), p-tau (**b**), SYP (**c**), PSD95 (**d**), AIF (**e**), Bcl-2 (**f**), Bax (**g**) and cleaved caspase-3 (**h**) in the hippocampus. (**C**) The protein expression images of Fpn1, TfR1, DMT1, GPX4 and DHODH in the hippocampus. (**D**) The protein expression level of Fpn1 (**a**), TfR1 (**b**), DMT1 (**c**), GPX4 (**d**) and DHODH (**e**) in the hippocampus. (**E**) The protein expression images of TLR4, NF-κB, HIF-1α, iNOS, VEGF and Glut1 in the hippocampus. (**F**) The protein expression level of TLR4 (**a**), NF-κB (**b**), HIF-1α (**c**), iNOS (**d**), VEGF (**e**) and Glut1 (**f**) in the hippocampus. (**A**,**C**,**E**) Control (a). AD (b). H-IN+Erastin (c). H-IG (d). L-IN (e). H-IN (f). ^###^ *p* < 0.001, ^##^ *p* < 0.01 vs. control group; * *p* < 0.05, ** *p* < 0.01, *** *p* < 0.001 vs. AD group; ^aa^ *p* < 0.01, ^aaa^ *p* < 0.001 vs. H-IN+Erastin group; ^b^ *p* < 0.05, ^bb^ *p* < 0.01 vs. H-IG group ($\bar{x}$ ± s, n = 10).

Based on the analysis of the Western blot and quantitative expression results (Figure 6A,B), the protein expression levels of tau, p-tau, AIF, Bax, and cleaved caspase-3 in the AD group of mice showed significant increases. Conversely, the expression levels of SYP, PSD95, and Bcl-2 were significantly decreased in this group. These findings indicate that the AD group mice exhibit marked pathological activation of tau along with notable apoptotic signaling and significant synaptic damage. Following treatment, the treatment group displayed substantial improvements in the abnormal expression of these proteins. Notably, the therapeutic effect of H-IN group was superior to that observed in the H-IG group, which was counteracted by Erastin. These results suggest that TF-Gel can effectively ameliorate the pathological expression of tau in AD, inhibit cellular apoptosis, and mitigate synaptic damage.

#### 3.8.2. Ferroptosis Regulatory Protein

To further investigate the regulatory role of TF-Gel in enhancing ferroptosis, we analyzed the expression of characteristic ferroptosis proteins, as illustrated in Figure 6C. In the AD group, we observed a significant reduction in the expression of Fpn1. Conversely, the expression levels of TfR1 and DMT1 were notably increased. Additionally, the protein expression levels of glutathione peroxidase GPX4 and DHODH were significantly lower, indicating that mice in the AD group displayed pronounced characteristics of ferroptosis. Figure 6D further quantifies the expression of these proteins. The H-IN group exhibited the most substantial effect in reversing the abnormal protein expression, while the H-IG group showed a similarly effective reversal, although to a lesser extent than the H-IN group. Furthermore, the reversal of the improvement effect of Erastin on TF-Gel substantiates at the molecular level that the inducer counteracts the anti-ferroptosis effect of the drug.

#### 3.8.3. TLR4/NF-κB/HIF-1α Signaling Pathway

To further analyze the mechanism by which TF-Gel improves AD, we conducted an in-depth examination of the expression levels of proteins involved in the TLR4/NF-κB/HIF-1α signaling pathway, as illustrated in Figure 6E,F. Mice in the AD group exhibited significant dysregulation in the expression of components associated with this signaling pathway, along with its downstream target proteins. Following treatment with TF-Gel, we observed a notable downregulation of TLR4, NF-κB, HIF-1α, and their downstream factor, iNOS, in the hippocampal tissue of the mice. In contrast, the expression levels of VEGF and Glut1 were significantly elevated. Compared to the H-IG group, these findings indicate that the H-IN group exhibited the most pronounced regulatory effect on the key nodes of this signaling pathway. Furthermore, the effects of TF-Gel on this pathway were entirely mitigated by Erastin, underscoring that TF-Gel inhibits ferroptosis in AD through the modulation of this signaling pathway.

## 4. Discussion

The pathological mechanisms of AD are extensive, exhibiting a high degree of complexity and systematicity, yet they are often simplified through unconventional single protein deposition hypotheses. Approaching these mechanisms exclusively through traditional pathways presents clear limitations in achieving meaningful breakthroughs [29,30]. Therefore, it is necessary to investigate the disease mechanism from novel perspectives in light of current research advancements. In recent years, the scope of investigation has broadened significantly, with ferroptosis and mitochondrial dysfunction emerging as two interrelated and mutually influential patterns of cellular homeostasis imbalance. These patterns have become central to frontier research aimed at understanding progressive neuronal loss [31,32]. The purpose of this study is to systematically explore the dynamic interaction between these two novel pathological links within a model of olfactory dysfunction-induced AD. Based on this exploration, we will evaluate whether a taxifolin-loaded thermosensitive hydrogel, utilizing a nasal delivery system, can effectively serve as a multi-target intervention strategy in AD treatment by coordinating this pathological axis, ultimately achieving neuroprotective effects.

Cognitive decline represents the most fundamental clinical manifestation of AD [33]. The significant enhancement in spatial learning and memory abilities observed in the Morris water maze experiment confirms the successful establishment of the OA-induced AD model. This finding provides compelling behavioral evidence for the effectiveness of TF-Gel in ameliorating AD symptoms. Following this, we conducted a histological examination of the hippocampus, a region closely associated with learning and memory. The H & E staining results demonstrated that the disordered arrangement and shrinkage necrosis of neurons in the CA1, CA3, and DG regions of the hippocampus improved markedly after intervention. Nissl staining further indicated that the observed improvements in hippocampal neurons corresponded to a significant recovery of the intracellular Nissl body structure. The consistency between water maze performance and histopathological findings provides preliminary evidence, from both behavioral and morphological perspectives, that taxifolin exerts significant neuroregulatory and protective effects in enhancing outcomes in AD.

In order to systematically elucidate the molecular mechanism of taxifolin in improving AD, this study focused on the core pathological characteristics of AD, combined with the screening results of network pharmacology and molecular docking technology. ELISA and WB techniques were used to systematically evaluate key pathways such as neuroinflammation, oxidative stress, and cell apoptosis. The significant increase in levels of pro-inflammatory cytokines such as IL-1β, IL-6, and TNF-α confirms the widespread activation of inflammatory responses. To trace its upstream regulatory nodes, we detected the activity of TLR4 and its downstream nuclear factor NF-κB signaling pathway. TLR4 is an important member of the toll-like receptor (TLR) family [34,35] and a key pattern recognition receptor in the innate immune system. It can respond to both exogenous invasion and endogenous damage in the body, and its overactivation further mediates immune dysfunction and inflammation in the body. In the process of AD, TLR4 can be activated by endogenous danger signals such as abnormally aggregated tau protein, thereby initiating its downstream pathway NF-κB, a nuclear factor-mediated inflammatory gene transcription program [36,37]. Therefore, intervening in the expression of TLR4 to connect the intracellular and extracellular environment can play a key role in regulating inflammation and immune homeostasis, which is a crucial step in the treatment of AD. Our research results indicate that this signaling pathway is significantly activated in the AD model, and taxifolin can effectively inhibit the abnormal expression of this signaling pathway during treatment. At the same time, the level of TGF-β1, which has anti-inflammatory and neuroprotective properties, was significantly upregulated in the treatment group, which helps to create a more favorable immune microenvironment for nerve repair.

We further investigated the role of HIF-1α in the TLR4/NF-κB signaling pathway. HIF-1α serves as a fundamental transcription factor for cellular adaptation to hypoxia and oxidative stress, and its mechanism of action is extensive [38,39]. This factor is regulated by various upstream signaling pathways, including the NF-κB pathway, while also being capable of simultaneously regulating multiple downstream genes, thereby integrating signals from nerve growth factors and oxidative stress. In the context of chronic inflammation and metabolic stress observed in AD, the stability of HIF-1α is continuously elevated [40,41]. This stabilization leads to the regulation of a series of downstream target genes. By targeting the crucial intermediate hub of HIF-1α, it may be possible to improve downstream inflammatory responses and oxidative stress, thereby mitigating related damage in addition to modulating the TLR4/NF-κB signaling pathway. The Western blot results demonstrate that the expression levels of TLR4, NF-κB, and HIF-1α in the hippocampal tissue of the AD model were significantly upregulated. Concurrently, the expression of their downstream target iNOS was markedly increased, whereas the levels of VEGF and Glut1 were significantly reduced. The downregulation of VEGF helps alleviate pathological angiogenesis in AD brain and improve blood–brain barrier integrity, thereby preventing peripheral inflammatory factors from entering the brain and reducing neuroinflammation [42,43]. The downregulation of Glut1 may help reverse abnormal glucose metabolism reprogramming in neurons, reduce oxidative stress and metabolic stress, and collectively reshape the microenvironment in the brain [44]. Additionally, ATP levels, which reflect cellular energy status, were considerably diminished. The content of the LDH showed a significant increase, indicating neuronal membrane damage and cell death [45]. Collectively, these alterations illustrate the phenomenon of “pseudo hypoxia” manifested by the AD model, whereby cellular metabolism shifts from efficient oxidative phosphorylation to inefficient glycolysis, resembling the “Warburg effect” typically observed in tumor biology [46]. Treatment with taxifolin exhibited a synergistic effect, inhibiting the expression of TLR4 and NF-κB as well as the activation of HIF-1α. This intervention also reversed the abnormal energy metabolism indicators mentioned earlier. Such a treatment strategy suggests the potential to disrupt the vicious cycle of inflammation and oxidative stress induced by metabolic disorders. By attenuating the metabolic disturbances driven by upstream neurodegenerative processes, this approach may provide an essential cellular microenvironment conducive to the subsequent repair of core organelles, particularly mitochondria, which are highly sensitive to energy and redox states. While this treatment strategy demonstrates promising multi-target effects, we acknowledge that direct causative validation of the TLR4/NF-κB/HIF-1α axis as the upstream trigger of ferroptosis would benefit from pathway-specific inhibitors (e.g., TAK-242 for TLR4) or genetic knockdown approaches. Nevertheless, the combination of network pharmacology predictions, molecular docking, and the functional link between pathway modulation and ferroptosis reversal (demonstrated by Erastin rescue) provides a strong foundation for the proposed mechanism. Future studies employing genetic and pharmacological interventions are warranted to further consolidate the hierarchical relationship between this signaling axis and ferroptosis in the context of AD.

Mitochondrial dysfunction is a critical pathological feature of AD [47,48]. This study systematically evaluated mitochondrial function and structure from multiple perspectives. As a key rate-limiting enzyme in the respiratory chain, the decreased activity of mitochondrial complex I serves as an early sensitive marker for damage to the electron transport chain [49,50]. Our results indicated that the activity of mitochondrial complex I in the hippocampus of AD patients was significantly reduced, whereas treatment with TF-Gel notably restored this activity. The excessive inhibition of complex I can lead to the increased production of ROS [51], a finding supported by observed changes in ROS expression as well as levels of MDA and SOD in this study. Additionally, the combined use of the JC-1 fluorescent probe with flow cytometry and fluorescence microscopy confirmed the severe depolarization of the mitochondrial membrane potential in AD neurons. However, TF-Gel effectively stabilized this membrane potential. These functional changes were corroborated at the ultrastructural level and TEM revealed typical pathological alterations in the mitochondria of the AD group, while significant improvements were noted in the treatment group. Restored mitochondrial function provides essential energy for synaptic activity. The Western blot analysis demonstrated the significant upregulation of synaptic proteins such as SYP and PSD95 following treatment, alongside a notable increase in the level of the key neurotrophic factor BDNF. Furthermore, abnormal levels of the excitatory neurotransmitter Glu in the hippocampus of AD patients may trigger synaptic imbalance and excitotoxicity [52,53], and taxifolin effectively regulates these abnormal Glu levels. BDNF enhances the resilience of neurons against Glu excitotoxicity and promotes the expression of synaptic proteins, thereby establishing a positive feedback loop that supports neural network repair. Functionally restored mitochondria also mitigate the release of apoptosis-inducing factors by stabilizing the outer mitochondrial membrane. This is consistent with an increased Bcl-2/Bax ratio and decreased expression of cleaved caspase-3, indicating the synergistic inhibition of mitochondrial pathway-mediated cell apoptosis. These findings suggest that taxifolin has established a multi-faceted protective network by enhancing mitochondrial function, regulating synaptic proteins and neurotransmitters, and inhibiting cell apoptosis.

Ferroptosis, a cell death pathway driven by iron-dependent lipid peroxidation, centers on the breakdown of the glutathione antioxidant system [54,55]. This study investigates the upstream driving factors associated with this process in AD. The abnormal expression of key iron homeostasis proteins, including Fpn1, TfR1, and DMT1, in AD neurons suggests a state of imbalance characterized by diminished iron output and increased input. This condition may lead to the accumulation of intracellular iron ions, thereby establishing a material basis for the occurrence of iron-dependent cell death. Moreover, the excessive reactive oxygen species produced by mitochondrial dysfunction can co-catalyze lipid peroxidation reactions involving iron. At the level of antioxidant defense, there is a noted downregulation of crucial ferroptosis inhibitory proteins such as GPX4 and mitochondrial DHODH [56,57], which reside in different cellular compartments. Concurrently, the activity of GSH-Px is significantly reduced, indicating a severe impairment in the overall ability of cells to repair lipid peroxidation. Intervention with TF-Gel has shown potential in correcting iron metabolism imbalances and increasing the expression of GPX4 and DHODH. Observations from electron microscopy further indicate that TF-Gel can ameliorate mitochondrial morphological abnormalities. Importantly, the administration of the ferroptosis inhibitor Erastin appears to negate the neuroprotective effects of taxifolin. This finding implies that the protective action of taxifolin is not confined to the direct or specific inhibition of the ferroptosis pathway. Rather, its effects may stem from a multi-target approach addressing upstream factors, including the inhibition of inflammation and oxidative stress, the repair of mitochondrial function, and the regulation of iron metabolism. This multifaceted approach systematically addresses the root causes driving ferroptosis, thereby establishing a mechanistic link between the repair of mitochondrial function and the inhibition of this programmed cell death pathway.

Furthermore, the efficacy observed in the positive control group receiving taxifolin via the traditional intragastric route was inferior to that of the TG-Gel group. This finding suggests that the temperature-sensitive hydrogel nasal delivery system employed in this study effectively facilitated the transport of taxifolin to the brain, maintaining therapeutic concentrations at the targeted site. By providing a non-invasive route for direct brain access, this innovative approach enables the realization of taxifolin’s inherent multi-target pharmacological potential, ultimately translating into significant central nervous system protective effects.

It is worth noting that the OA-induced model employed in this study primarily recapitulates the tau pathology and associated neurodegeneration, which are core features of AD, while not exhibiting amyloid-β deposition. Given that our investigation focused on tau-mediated mitochondrial dysfunction and ferroptosis, this model provides a relevant and well-established platform for mechanistic exploration. Nevertheless, to further consolidate the therapeutic potential of TF-Gel across the full spectrum of AD pathology, ongoing studies in our laboratory are extending these findings to chronic and transgenic AD models (e.g., APP/PS1 and 3xTg-AD), which will provide complementary insights into its efficacy in the presence of both tau and amyloid pathologies.

## 5. Conclusions

In this study, we investigated the therapeutic effects of a thermosensitive hydrogel carrying taxifolin in an OA-induced AD model. The findings demonstrated that this delivery system significantly improved the underlying pathological mechanisms of AD by modulating the TLR4/NF-κB/HIF-1α axis through efficient nasal delivery to the brain. The treatment ameliorated pathological damage to nerve tissue, reduced neuroinflammation, facilitated the clearance of ROS, and ameliorated oxidative stress damage. At both ultrastructural and molecular biological levels, our results provide evidence that taxifolin synergistically enhances mitochondrial function, restores iron homeostasis in nerve cells, and effectively inhibits lipid peroxidation and ferroptosis by regulating the expression of the key proteins involved in these processes. Consequently, it protects the structure and function of neurons through a multifaceted approach. This study establishes a new experimental basis for the use of taxifolin in the synergistic treatment of AD, highlighting multiple targets and pathways that could be potentially exploited for therapeutic intervention.

## Figures and Tables

**Table 1:** Molecular docking results of taxifolin with core target protein.

Target Protein	PDB ID	Active Ingredient	Binding Energy (kcal/mol)
TLR4	4G8A	Taxifolin	−6.9
HIF1A	4H6J		−6.3
PTGS2	5F19		−10.0
NFKB1	1SVC		−6.7
MAPK1	2Y9Q		−9.0

## Data Availability

The datasets analyzed in this study were obtained from publicly available databases. These data were derived from the following resources available in the public domain: PubChem: https://pubchem.ncbi.nlm.nih.gov/; SuperPred: https://prediction.charite.de/subpages/target_prediction.php; TCMSP: https://tcmspw.com/tcmsp.php; GeneCards: https://www.genecards.org/; OMIM: https://www.omim.org/; STRING: https://string-db.org/; RCSB PDB: http://www.rcsb.org/ (accessed on 15 June 2025).

## Abbreviations

The following abbreviations are used in this manuscript:

AD	Alzheimer’s disease
TF	Taxifolin
TF-Gel	Taxifolin-loaded thermosensitive hydrogel
OA	Okadaic acid
ROS	Reactive oxygen species
Aβ	β-amyloid protein
NIPAM	N-isopropylacrylamide
TEMED	Tetramethylethylenediamine
KPS	Potassium persulfate
PVP-K30	Polyvinylpyrrolidone
GelMA	Methacrylated gelatin
Venn	Venn diagram
PPI	Protein interaction network
GO	Gene Ontology Analysis
KEGG	Kyoto Encyclopedia of Genes and Genomes
BP	Biological process
CC	Cellular component
MF	Molecular function
MWM	Morris water maze
SOD	Superoxide dismutase
IL-1β	Interleukin-1β
IL-6	Interleukin-6
TNF-α	Tumor necrosis factor-alpha
MDA	Malondialdehyde
NADPH	Nicotinamide adenine dinucleotide phosphate
GSH-Px	Glutathione peroxidase
BDNF	Brain-derived neurotrophic factor
TGF-β	Transforming growth factor-beta
Glu	Glutamic acid
LDH	Lactate dehydrogenase
ATP	Adenosine triphosphate
AOD	Average optical density
TEM	Transmission electron microscope
MMP	Mitochondrial membrane potential
WB	Western blot
SYP	Synaptophysin
PSD95	Post synaptic density protein 95
AIF	Apoptosis-inducing factor
Fpn1	Ferroportin 1
TfR1	Transferrin receptor 1
DMT1	Divalent metal transporter 1
GPX4	Glutathione peroxidase 4
DHODH	Dihydroorotate dehydrogenase
TLR4	Toll-like receptor 4
NF-κB	Nuclear factor kappa-B
HIF-1α	Hypoxia-inducible factor 1-alpha
iNOS	Inducible nitric oxide synthase
VEGF	Vascular endothelial growth factor
Glut1	Glucose transporter 1
SDS-PAGE	Sodium dodecyl sulfate polyacrylamide gel electrophoresis
PVDF	Polyvinylidene fluoride
TBS	Tween-20 tris Buffered saline
ECL	Enhanced chemiluminescence
SD	Standard deviation
